# Investigating multidimensional associations between obesity-related traits and brain health, and identifying potential mechanisms

**DOI:** 10.1097/MD.0000000000046831

**Published:** 2026-01-09

**Authors:** Shuyuan Chen, Jie Wen, Zeming Tan, Yuyao Chen, Zhiwei Xia, Hongwei Liu

**Affiliations:** a Department of Pediatrics, Xiangya Hospital, Central South University, Changsha, China; b Department of Neurosurgery, Xiangya Hospital, Central South University, Changsha, China; c National Clinical Research Center for Geriatric Disorders, Xiangya Hospital, Central South University, Changsha, China; d Department of Epidemiology and Health Statistics, Xiangya School of Public Health, Central South University, Changsha, Hunan Province, China; e Department of Neurology, Hunan Aerospace Hospital, Hunan Normal University, Changsha, China.

**Keywords:** brain health, Mendelian randomization, NHANES, obesity, TWAS

## Abstract

The impact of obesity on brain health, spanning cerebrovascular disease, cognition, and neuroanatomy, remains incompletely understood. We conducted 2-sample Mendelian randomization to assess the causal effects of obesity traits on brain health outcomes. Potential biological pathways were explored using transcriptome-wide association study analyses. We further examined associations in the National Health and Nutrition Examination Survey using weighted, multivariable-adjusted logistic regression. Obesity measures included body mass index (BMI), waist circumference (WC), and waist-to-hip ratio (WHR). Outcomes comprised stroke, fluid intelligence, and brain image-derived phenotypes. Higher BMI (odds ratio [OR] = 1.18; 95% confidence interval [CI], 1.13–1.24; *P* = 8.99 × 10^−12^), WC (OR = 1.20; 95% CI, 1.09–1.31; *P* = 3.85 × 10^−3^), and WHR (OR = 1.23; 95% CI, 1.16–1.31; *P* = 9.19 × 10^−11^) were associated with increased stroke risk. BMI (β = −0.21; *P* = 8.62 × 10^−6^) and WHR (β = −0.33; *P* = 1.19 × 10^−7^) were linked to lower fluid intelligence scores. BMI showed causal effects on 7 brain image-derived phenotypes. Transcriptome-wide association study implicated pathways related to substrate metabolism, immune activation, and epigenetic regulation in mediating obesity’s effects on brain health. In National Health and Nutrition Examination Survey, after adjustment for covariates, BMI (OR = 1.04; 95% CI, 1.02–1.06; *P* = 1.27 × 10^−3^), WC (OR = 1.01; 95% CI, 1.00–1.02; *P* = 9.58 × 10^−3^), and WHR (OR = 1.40; 95% CI, 1.08–1.81; *P* = 1.25 × 10^−2^) were positively associated with stroke risk. Obesity, particularly BMI, shows causal relationships with stroke risk, alterations in brain structure, and reduced cognitive performance. Mechanisms may involve metabolic, immune, and epigenetic pathways. These findings underscore the importance of obesity prevention and management to preserve brain health.

## 1. Introduction

Following the development of the global economy and higher living standards, the prevalence of overweight and obesity has doubled over the past 50 years.^[1]^ The NCD Risk Factor Collaboration data published in *The Lancet* indicate a significant increase in the prevalence of obesity in children and adolescents from 1975 to 2016. Specifically, during this period, the prevalence of obesity in boys rose from 0.7% to 5.6%, while in girls, it increased from 0.9% to 7.8%.^[2]^ The prevalence of obesity among adults has also shown a major growth across the world, with the age-standardized incidence rate of obesity in men rising from 3.2% in 1975 to 10.8% in 2014 and in women increasing from 6.4% in 1975 to 14.9% in 2014.^[3]^ Obesity has emerged as one of the major issues threatening global public health. Obese individuals are at an elevated risk of cardiovascular disease, type 2 diabetes mellitus, hypertension, and certain cancers.^[4]^ Nevertheless, the influence of obesity on cerebral health remains incompletely understood.

Growing evidence suggests that obesity can increase the risk of neurodegenerative diseases such as Alzheimer disease.^[5]^ A higher prevalence of brain function and structure changes has been reported in the obese population.^[6]^ A systematic review and meta-analysis study revealed that the total brain volume was negatively correlated with body mass index (BMI) and waist circumference (WC). Moreover, gray matter volume was adversely associated with BMI, and hippocampal volume with BMI, WC, and waist-to-hip ratio (WHR).^[7]^ Another systematic review of cross-sectional studies suggested an adverse association between obesity and cognition between the ages of 19 and 65.^[8]^ The pathogenesis of obesity on brain health is currently incompletely understood, primarily involving inflammatory factors, hormone levels, metabolites, and cerebrovascular dysfunction.^[9]^

In contrast to the presence of confounding factors in observational studies and the difficulty in determining causal relationships, Mendelian randomization (MR) analysis provides a suitable approach. Various MR studies have been conducted on obesity and brain health, which generally investigated a particular aspect of the nervous system, lacking a global perspective. Considering the complex impact of obesity on the brain, our study analyzed the overall causal effect of obesity on brain health using MR, including brain diseases, cognition, and brain structure. Subsequently, transcriptome-wide association study (TWAS) was conducted to identify potential signaling pathways mediating the effects of obesity on brain health. Finally, the relationship between obesity and brain diseases was analyzed by retrospective analyses on the National Health and Nutrition Examination Survey (NHANES) 2017 to 2020 dataset.

## 2. Methods

### 2.1. Overall research workflow

**Figure 1.**
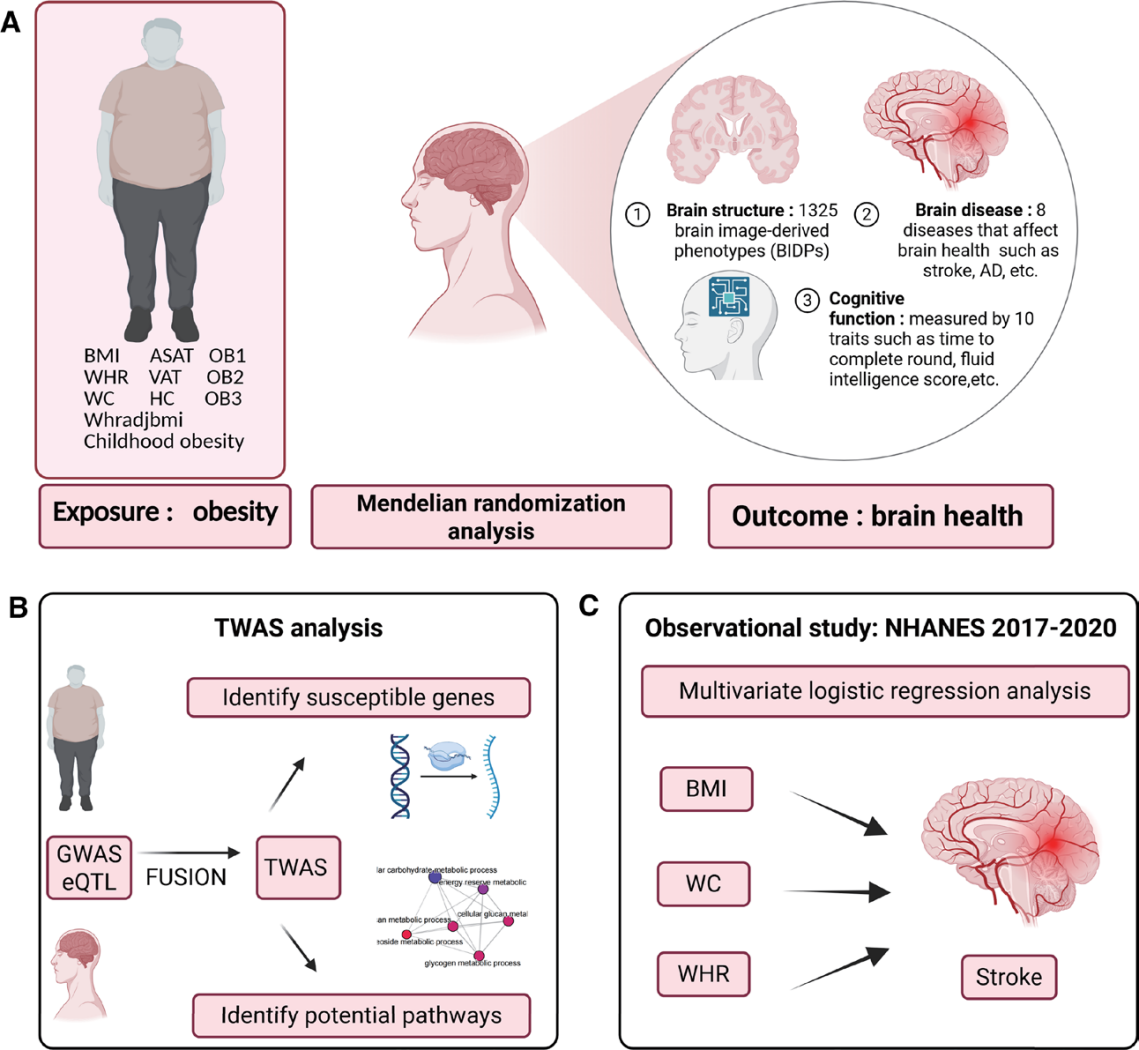
Schematic diagram of the research process. Mendelian randomization study between obesity and brain health (A); exploring susceptible genes and potential pathways between obesity and brain health by TWAS (B); observational study on obesity and brain diseases by multivariate logistic regression analysis in NHANES 2017 to 2020 database (C). NHANES = National Health and Nutrition Examination Survey, TWAS = transcriptome-wide association study. Figure 1 was built by the Biorender (Agreement number: KF292OWC0Q, https://BioRender.com/y889gc3).

This study consists of 3 parts. Part 1: MR analysis was conducted on genome-wide association study (GWAS) data to explore the causal relationship between obesity and brain health. Part 2: Potential signaling pathways between obesity and brain health were examined by TWAS. Part 3: Retrospective analyses were conducted on NHANES datasets, utilizing multivariate regression analysis to validate the relationship between obesity and brain disorders. The overall process is shown in Figure 1.

## 3. Mendelian randomization

### 3.1. Exposure data

Obesity was selected as an exposure factor and was measured by abdominal subcutaneous adipose tissue volume (ASAT), visceral adipose tissue volume (VAT), BMI, WHR, waist-hip ratio adjusted for BMI, WC, hip circumference, obesity class 1, obesity class 2, obesity class 3, and childhood obesity. GWAS summary statistics for abdominal subcutaneous adipose tissue volume and VAT were downloaded from the study of Liu et al.^[10]^ GWAS summary statistics for BMI, WHR, and waist-hip ratio adjusted for BMI were obtained from the research of Pulit, SL et al,^[11]^ which included 694,649 participants. GWAS summary statistics for WC, hip circumference, obesity class 1, obesity class 2, and obesity class 3 were downloaded from Genetic Investigation of Anthropometric Traits,^[12,13]^ and childhood obesity from Early Growth Genetics Consortium.^[14]^ Further details are presented in Table S1, Supplemental Digital Content, https://links.lww.com/MD/R43.

## 4. Outcome data

The outcome factors included 3 aspects: brain diseases, cognition, and brain structure. A total of 8 diseases affecting brain health were selected as outcomes, including stroke,^[15]^ Alzheimer dementia,^[16]^ amyotrophic lateral sclerosis,^[17]^ brain glioblastoma and astrocytoma, multiple sclerosis,^[18]^ benign neoplasm of the brain and other parts of the central nervous system, dementia with Lewy bodies,^[19]^ and neuroticism sum score.^[20]^ Cognition was measured by 10 traits, including time to complete the round, the number of correct symbol digit matches, the duration to complete the numeric path, the mean time to correctly identify matches, the number of symbol digit matches attempted, the maximum number of digits remembered correctly, the duration to complete the alphanumeric path, the number of incorrect matches in the round, the prospective memory result, and the fluid intelligence score. The data regarding brain structure encompassed 1325 brain image-derived phenotypes (BIDPs) sourced from the UK Biobank. The data comprised 647 magnetic resonance imaging phenotypes related to brain regional and tissue volume, including 372 pertaining to the cortical area and 306 associated with cortical thickness.^[21]^ Detailed information on the GWAS data of these outcome factors is listed in Table S1, Supplemental Digital Content, https://links.lww.com/MD/R43.

## 5. Instrumental variable selection

Obesity was selected as the exposure, while brain health was set as the outcome. First, Single Nucleotide Polymorphisms (SNPs) significantly correlated with 11 traits of obesity were selected as IVs. A threshold of *P* < 1 × 10^−7^ was set to select significant SNPs for 5 traits (WHR, whradbmi, WC, BMI, and VAT) related to obesity, while a threshold of *P* < 1 × 10^−6^ was used for the other 6 traits. Second, the clumping process (*R*^2^ < 0.01 and clumping distance = 10,000 kb) was conducted to eliminate the linkage disequilibrium among the included SNPs. Subsequently, the effect SNPs were extracted from the outcome database to ensure their corresponding effects on the outcome. Finally, palindromic SNPs were deleted.

### 5.1. Transcriptome-wide association study

A quantitative trait loci-based linear model was trained using reference panels from Genotype-Tissue Expression version 8 (N = 558).^[22]^ In addition, the training model was utilized to predict gene expression, and GWAS data were converted into TWAS data.^[23]^ The TWAS outcomes consistently revealed genes that were significantly linked with both obesity and brain health. These genes were subjected to biological pathway enrichment analysis to investigate the underlying biological mechanisms. The *P*-values for obesity and brain health were combined using Fisher combined *P*-value method, providing a comprehensive understanding of the associations.

### 5.2. The observational study in the NHANES database

**Figure 2.**
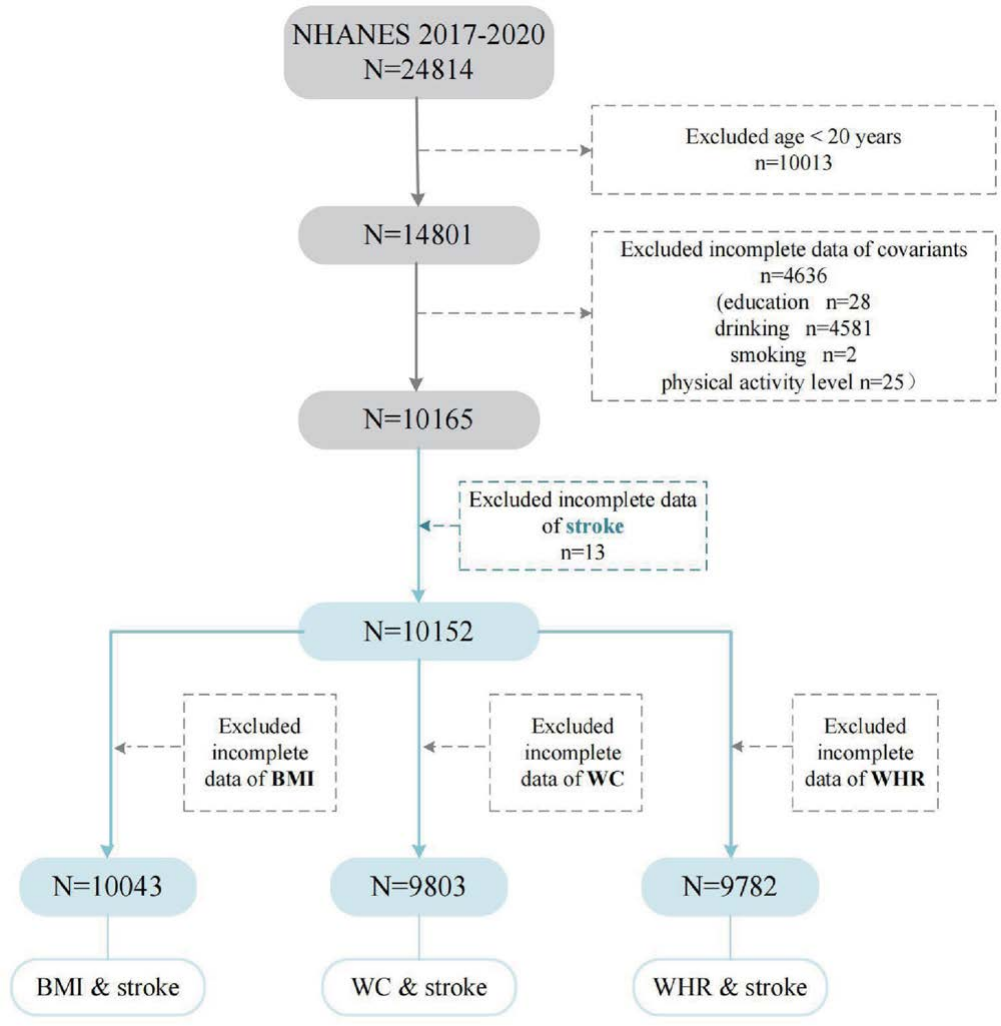
The screening process for participants in the NHANES. NHANES = National Health and Nutrition Examination Survey.

The study population consisted of participants from NHANES from 2017 to 2020 (N = 24,814). The ethics committee has approved the research protocol of NHANES, and all NHANES participants have provided informed consent. The exclusion criteria included the following: age < 20 years (N = 10,013); incomplete data of covariates (N = 4636, education n = 28, drinking n = 4581, smoking n = 2, physical activity level n = 25); and incomplete information on the main variable. The overall screening process is shown in Figure 2. Multivariate-adjusted logistic regression was performed to assess the relationship between obesity indicators and brain diseases. Three models were evaluated with adjusted covariates: Model 1 was not adjusted, Model 2 was adjusted for sex, age, race, and education, and Model 3 was further adjusted for sex, age, race, education, drinking, smoking, and physical activity.

### 5.3. Statistical analysis

MR analysis was conducted to investigate the causality between obesity and brain health using the inverse variance weighting (IVW) method,^[24]^ the weighted median method,^[25]^ and MR-Egger,^[26]^ as previously described.^[24,25]^ Furthermore, MR-Egger intercept tests were conducted to assess the robustness of the results.^[26]^ Lastly, heterogeneity testing was performed using Cochran Q test.^[27]^ Multivariable logistic regression analysis was conducted to explore the association between obesity and brain diseases. Odds ratios (OR) and 95% confidence intervals (CIs) were calculated to assess the strength of this association. A *P*-value < .05 was regarded as statistically significant.

## 6. Results

### 6.1. The causal effects of obesity on brain health

**Figure 3.**
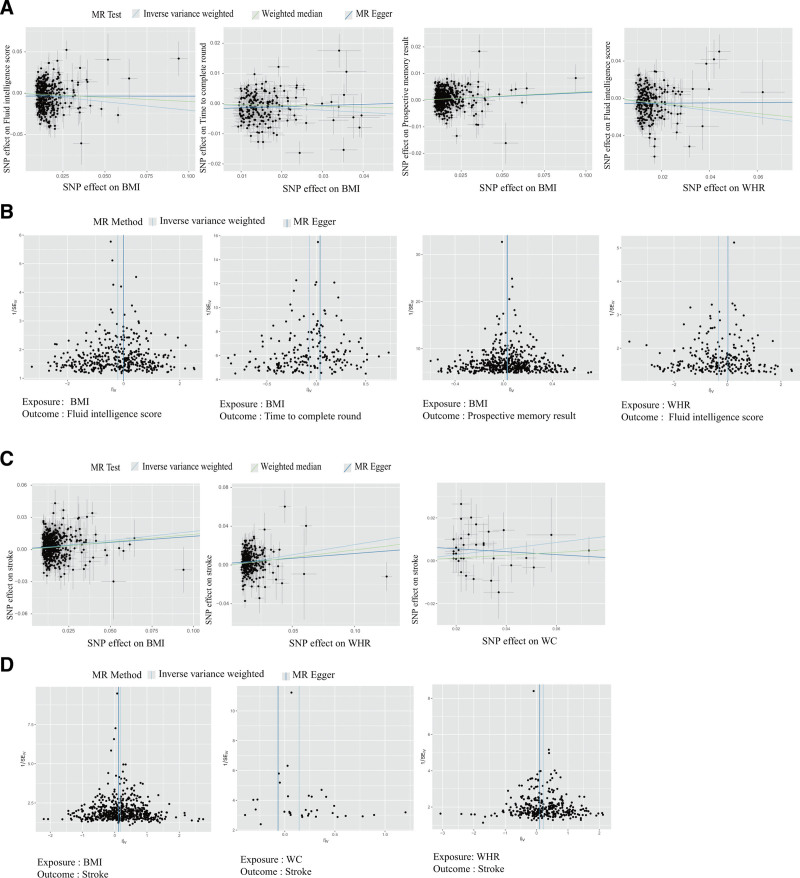
The scatter plot and funnel plot of the causal effect between obesity and brain health. The scatter plot (A) and funnel plot (B) of the association between obesity and cognition; the scatter plot (C) and funnel plot (D) of the association between obesity and brain disease.

First, MR analysis was performed to investigate the relationship between obesity and 8 brain diseases. Based on the IVW estimate, BMI (OR = 1.18; 95% CI: 1.13–1.24; *P* = 8.99 × 10^−12^), WC (OR = 1.20; 95% CI: 1.09–1.31; *P* = 3.85 × 10^−3^), and WHR (OR = 1.23; 95% CI: 1.16–1.31; *P* = 9.19 × 10^−11^) were identified to increase the risk of stroke. Second, the causal relationship between obesity and cognition was explored. The IVW method revealed that BMI and WHR were causally associated with cognition. Moreover, BMI (β = −0.21, *P* = 8.62 × 10^−6^) and WHR (β = −0.33, *P* = 1.19 × 10^−7^) showed a causal association with lower fluid intelligence score. In addition, BMI was shown to decrease the time to complete the round (β = −0.07, *P* = 2.18 × 10^−4^) while increasing prospective memory results (β = 0.03, *P* = 4.31 × 10^−5^). Finally, the causal relationship between obesity and brain structure was analyzed. BIDPs are unique measures of brain structure derived from raw magnetic resonance imaging data. MR analysis was conducted to investigate the causal relationship between obesity and BIDPs. The results of IVW method indicated a causal association between BMI and increased levels of 7 BIDPs: IDP 165 (β = 0.16, *P* = 7.95 × 10^−8^), IDP 166 (β = 0.15, *P* = 1.27 × 10^−6^), IDP 170 (β = 0.15, *P* = 5.22 × 10^−7^), IDP 187 (β = 0.18, *P* = 8.86 × 10^−10^), IDP 648 (β = 0.15, *P* = 5.00 × 10^−7^), IDP 682 (β = 0.15, *P* = 1.95 × 10^−6^), and IDP 716 (β = 0.15, *P* = 1.66 × 10^−6^). Additionally, a sensitivity analysis was performed. The MR-Egger regression intercept analysis was employed to investigate horizontal pleiotropy, revealing no evidence of horizontal pleiotropy. The detailed results of the MR analysis between obesity and brain health are listed in Tables 1 and 2. The scatter plot and funnel plot are shown in Figure 3 and Figure S1, Supplemental Digital Content, https://links.lww.com/MD/R44. The leave-one-out plot is illustrated in Figures S2–S4, Supplemental Digital Content, https://links.lww.com/MD/R44.

**Table 1:** The MR analysis of obesity and brain disease, and its sensitivity analysis.

Exposure	nSNP	OR	LCI	HCI	IVW-*P*val	Q_*P*val	intercept-*P*val	Outcome
BMI	538	1.18	1.13	1.24	8.99 × 10^−12^	1.26 × 10^−14^	.36	Stroke
WHR	344	1.23	1.16	1.31	9.19 × 10^−11^	6.21 × 10^−13^	.19	Stroke
WC	34	1.20	1.09	1.31	3.85 × 10^−3^	6.66 × 10^−2^	.06	Stroke

BMI = body mass index, HCI = higher confidence interval, IVW = inverse variance weighting, LCI = lower confidence interval, MR = Mendelian randomization, OR = odds ratios, WC = waist circumference, WHR = waist-to-hip ratio.

**Table 2:** The MR analysis of obesity and cognition, brain structure, and its sensitivity analysis.

Exposure	nSNP	β	SE	IVW-*P*val	Q_*P*val	intercept-*P*val	Outcome
BMI	398	−0.21	4.65 × 10^−2^	8.62 × 10^−6^	1.61 × 10^−72^	.10	Fluid intelligence score
BMI	203	−0.07	1.96 × 10^−2^	2.18 × 10^−4^	4.31 × 10^−52^	.07	Time to complete round
BMI	539	0.03	7.56 × 10^−3^	4.31 × 10^−5^	2.78 × 10^−25^	.88	Prospective memory result
WHR	248	−0.33	6.20 × 10^−2^	1.19 × 10^−7^	1.22 × 10^−51^	.07	Fluid intelligence score
BMI	475	0.16	3.04 × 10^−2^	7.95 × 10^−8^	2.53 × 10^−10^	.94	IDP 165
BMI	475	0.15	3.02 × 10^−2^	1.27 × 10^−6^	9.65 × 10^−10^	.92	IDP 166
BMI	475	0.15	2.95 × 10^−2^	5.22 × 10^−7^	1.63 × 10^−7^	.54	IDP 170
BMI	474	0.18	2.88 × 10^−2^	8.86 × 10^−10^	1.38 × 10^−5^	.89	IDP 187
BMI	474	0.15	3.07 × 10^−2^	5.00 × 10^−7^	8.69 × 10^−11^	.57	IDP 648
BMI	474	0.15	3.07 × 10^−2^	1.95 × 10^−6^	5.30 × 10^−11^	.58	IDP 682
BMI	475	0.15	3.14 × 10^−2^	1.66 × 10^−6^	7.93 × 10^−14^	.36	IDP 716

BMI = body mass index, IVW = inverse variance weighting, MR = Mendelian randomization, SE = standard error, WHR = waist-to-hip ratio.

### 6.2. Identification of the possible pathways by which obesity exerts its influence on brain health

**Figure 4.**
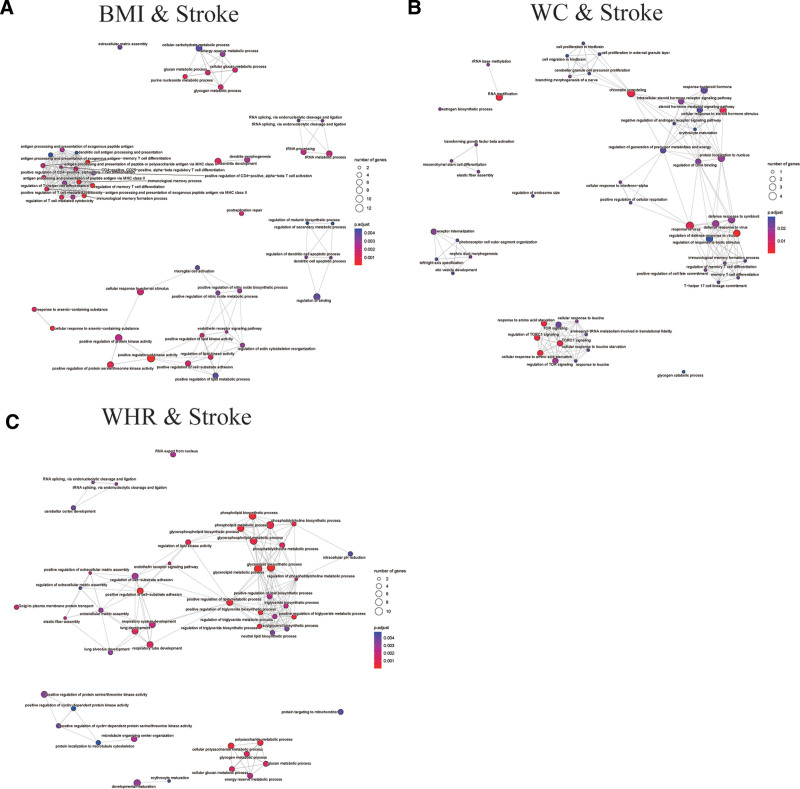
Biological pathways significantly enriched between obesity and stroke by TWAS. TWAS = transcriptome-wide association study.

The TWAS was performed to identify genes significantly associated with obesity and brain health. Additionally, biological pathway enrichment analysis was conducted to identify potential pathway mechanisms. A total of 250, 192, and 215 biological pathways were significantly enriched in BMI and IDP 165, IDP 166, and IDP 170, respectively (Fig. S5, Supplemental Digital Content, https://links.lww.com/MD/R44). These pathways are primarily involved in the metabolic processes of carbohydrates, fatty acids, proteins, as well as hormones and epigenetic regulations of protein modification. Moreover, 218, 227, and 164 biological pathways were significantly enriched in BMI and IDP 648, IDP 682, and IDP 716, respectively. These pathways are mainly involved in metabolic processes, epigenetic regulations, cell migration, and immune activities (Fig. S5, Supplemental Digital Content, https://links.lww.com/MD/R44). Furthermore, 287, 99, and 209 biological pathways were significantly enriched in BMI, WC, WHR, and stroke, respectively (Fig. 4). The pathways between BMI, WHR, and stroke were mainly involved in metabolic processes, cytoskeleton regulation, cell adhesion, cell migration, and neural development. In addition, the pathways between WC and stroke were mainly involved in the cellular response to various substances and immune activities. A total of 151 and 215 biological pathways were significantly enriched in BMI, WHR, and fluid intelligence scores, which were primarily involved in epigenetic regulation, neural development, and cytoskeleton regulation (Fig. S6, Supplemental Digital Content, https://links.lww.com/MD/R44). The significantly enriched biological pathways related to obesity and brain health are listed in Tables S2–S15, Supplemental Digital Content, https://links.lww.com/MD/R43.

### 6.3. Observational associations between obesity and brain diseases in NHANES

In the MR analysis, the causal relationship between obesity and 8 brain diseases was analyzed, revealing that 3 traits of obesity (BMI, WC, and WHR) were causally related to stroke. Furthermore, the correlation between the 3 traits and stroke was validated by multivariate regression analysis of data from the NHANES database. A total of 24,814 participants were enrolled in the NHANES database from 2017 to 2020. After excluding individuals under the age of 20 and those lacking covariate information (education, drinking, smoking, and physical activity level), 10,165 individuals were included for analysis. Characteristics of the study participants are listed in Table 3. After adjusting for the covariates in Model 3, the results indicated that BMI (OR = 1.04; 95% CI: 1.02–1.06; *P* = 1.27 × 10^−3^), WC (OR = 1.01; 95% CI: 1.00–1.02; *P* = 9.58 × 10^−3^), and WHR (OR = 1.40; 95% CI: 1.08–1.81; *P* = 1.25 × 10^−2^) were positively associated with stroke. The conclusions of our observational study were consistent with the MR analysis. The results of multivariate regression analysis are listed in Table 4.

**Table 3:** Characteristics of the study participants in the NHANES.

Characteristics	Overall
Sex (%)	
Female	5164 (51.4)
Male	4879 (48.6)
Age (median [IQR])	48.0 [33.0, 62.0]
Race (%)	
Mexican American	1268 (12.6)
Non-Hispanic Black	2541 (25.3)
Non-Hispanic White	3464 (34.5)
Other Hispanic	975 (9.7)
Other race	1795 (17.9)
Education (%)	
<9th grade	647 (6.4)
9–11th grade	968 (9.6)
High school graduate/GED or equivalent	2346 (23.4)
Some college or AA degree	3426 (34.1)
College graduate or above	2656 (26.4)
Drinking (%)	
Never	1213 (12.1)
Mild	4531 (45.1)
Moderate	2078 (20.7)
Heavy	2221 (22.1)
Smoking (%)	
Never	5951 (59.3)
Former	2201 (21.9)
Now	1891 (18.8)
PA level (%)	
Sedentary	2254 (22.4)
Low	1339 (13.3)
Middle	977 (9.7)
High	5473 (54.5)
BMI (median [IQR])	28.7 [24.8, 33.6]
WC (median [IQR])	99.0 [88.4, 111.0]
WHR (mean (SD))	0.9 (0.1)
Stroke (%)	
No	9647 (96.1)
Yes	396 (3.9)

AA = associate of arts, BMI = body mass index, GED = general educational development, IQR = interquartile range, NHANES = National Health and Nutrition Examination Survey, PA = physical activity, WC = waist circumference, WHR = waist-to-hip ratio.

**Table 4:** Association between obesity and brain disease in the NHANES.

Obesity traits	Brain disease	Model 1		Model 2		Model 3	
		OR (95% CI)	*P*-value	OR (95% CI)	*P*-value	OR (95% CI)	*P*-value
BMI	Stroke	1.03 (1.01,1.04)	1.00 × 10^−3^	1.03 (1.01,1.05)	4.21 × 10^−3^	1.04 (1.02,1.06)	1.27 × 10^−3^
WHR	Stroke	1.75 (1.44,2.13)	1.00 × 10^−4^	1.41 (1.09,1.82)	1.07 × 10^−2^	1.40 (1.08,1.81)	1.25 × 10^−2^
WC	Stroke	1.02 (1.01,1.03)	1.00 × 10^−4^	1.01 (1.00,1.02)	2.58 × 10^−2^	1.01 (1.00,1.02)	9.58 × 10^−3^

CI = confidence interval, BMI = body mass index, NHANES = National Health and Nutrition Examination Survey, OR = odds ratio, WC = waist circumference, WHR = waist-to-hip ratio.

## 7. Discussion

Our study conducted a comprehensive MR analysis on obesity and brain health, encompassing 3 aspects: brain diseases, cognition, and brain structure. Among the 11 traits of obesity, BMI showed the most extensive causal relationship with brain health. BMI was found to increase the risk of stroke and affect cognition. Moreover, a causal correlation was found between BMI and 7 BIDPs for the brain structure. Similarly, WHR can increase the risk of stroke and reduce fluid intelligence scores. However, no significant causal association was observed between WHR and brain structure. WC was found to increase the risk of stroke while exerting no causal effect on cognition or brain structure. Conversely, the other 8 traits of obesity demonstrated no statistically significant causal effects on brain health. In our observational cross-sectional study on the NHANES 2017 to 2020 dataset, stroke was positively correlated with BMI, WHR, and WC.

A meta-analysis of observational and MR studies between BMI and stroke showed that higher BMI was associated with all-cause stroke in the observational study; however, the MR study revealed no causal effect.^[28]^ The conclusions of observational studies were the same as ours, but the results of the MR analysis were different. This meta-analysis of the MR study included populations of different ethnicities, whereas our study only included the European population, which may lead to population bias. The Norwegian HUNT study found that obesity was a high-risk factor for ischemic stroke, but obese individuals with normal metabolism had a similar risk of ischemic stroke as those with normal weight individuals.^[29]^ An animal study revealed that obese mice had a higher mortality rate, more severe cerebral infarction, and edema after stroke compared with lean mice. This was related to the increased expression of VEGF-A and VEGFR2.^[30]^ Future cellular and molecular research may provide insights into the intrinsic pathogenesis between obesity and stroke.

Numerous studies have investigated the effects of obesity on cognition and brain structure. A population-based cohort study in Asia revealed an independent relationship between obesity and cognition. The results of MR analysis support a causal association between BMI and cognition.^[31]^ In contrast, another MR study in the UK Biobank suggests no causal relationship between obesity and cognition.^[32]^ Various studies have shown inconsistent results, which may be related to the differences in ethnic groups and the disparities in methods used to evaluate cognition. In the present MR analysis, cognition was evaluated by ten traits. On the one hand, BMI and WHR demonstrated a causal association with reduced fluid intelligence score; on the other hand, BMI exhibited a causal association with decreased time to complete rounds and increased prospective memory results. These findings highlight the complexity of the relationship between obesity and cognitive function.

Numerous studies have investigated the impact of obesity on brain structure. A meta-analysis of cross-sectional studies revealed that BMI was negatively correlated with total brain volume and gray matter volume.^[7]^ In an MR study, the outcomes demonstrated a positive causal connection between BMI and the cortical surface area of the transverse temporal, as well as between WHR and the cortical surface area of the isthmus cingulate. However, a negative causal relationship was observed between WHR and the cortical surface area of the inferior temporal.^[33]^ This study found a positive causal relationship between BMI and 7 BIDPs, mainly regarding the volume and area of specific brain regions. Nonetheless, the causal relationship between BMI and the thickness of brain regions was not statistically significant. Moreover, the other 10 traits of obesity exerted no statistically significant impact on brain structure in our study. The MR analysis of the effects of obesity on brain structure varied depending on different database sources of exposure and outcomes, as well as confounding factors such as sample age.

Some studies suggested that obesity-induced inflammation and metabolic disorders could lead to dysfunction of the blood-brain barrier and cause neurological disorders.^[34,35]^ Peripheral visceral adipose tissue has been proven to promote macrophages to enter the hypothalamus.^[36]^ A study has shown that obesity can affect methylation in the hippocampus, thereby altering gene expression related to neurodegeneration and metabolism.^[37]^ This was consistent with our TWAS results, which mainly affected metabolism, epigenetics, and immune activation.

Although our study is the most comprehensive research on obesity and brain health to date, the limitations should be acknowledged. Our databases were all sourced from Europe, which may result in population racial bias. The impact of obesity on brain health may vary among different age groups, but subgroup analysis based on age was not conducted in the present research. The classification of brain diseases could be further refined; for example, stroke could be further divided into ischemic stroke and hemorrhagic stroke to enhance research accuracy. In this MR analysis, Cochran Q test revealed significant heterogeneity. However, the random-effects IVW approach was employed to ensure the reliability of the results. In the future, more refined research should be carried out to elucidate the relationship between obesity and brain health.

## 8. Conclusions

This research combined MR analysis, TWAS, and NHANES retrospective analysis to comprehensively explore the impact of obesity on brain health, encompassing 3 key aspects: brain diseases, cognition, and brain structure. MR analysis was conducted between 11 obesity traits and 8 brain diseases, revealing a causal relationship between BMI, WC, WHR, and stroke. The same correlation was confirmed in the subsequent retrospective analysis in the NHANES database. In addition, a causal relationship was observed between BMI and cognition, as well as 7 BIDPs. The TWAS results indicated that the pathways mediating the effects of obesity on brain health were primarily involved in substance metabolism, immune activation, and epigenetic regulation. In-depth research on the pathological mechanisms underlying the relationship between obesity and brain health will be a future research direction.

## Acknowledgments

The authors express gratitude to the public databases, websites, and software used in the paper. The authors are grateful to the High-Performance Computing Center of Central South University for partial support of this work.

## Author contributions

**Conceptualization:** Hongwei Liu.

**Data curation:** Jie Wen, Yuyao Chen, Zhiwei Xia.

**Funding acquisition:** Shuyuan Chen, Hongwei Liu.

**Methodology:** Shuyuan Chen, Jie Wen, Zeming Tan.

**Project administration:** Hongwei Liu.

**Supervision:** Hongwei Liu.

**Validation:** Jie Wen, Yuyao Chen, Zhiwei Xia, Hongwei Liu.

**Visualization:** Hongwei Liu.

**Writing – original draft:** Shuyuan Chen.

**Writing – review & editing:** Shuyuan Chen, Jie Wen, Zeming Tan, Yuyao Chen, Zhiwei Xia, Hongwei Liu.

## Supplementary Material





## References

[1] Chooi YC Ding C Magkos F. The epidemiology of obesity. Metabolism. 2019;92:6–10. 30253139 10.1016/j.metabol.2018.09.005

[2] Collaboration NCDRF. Worldwide trends in body-mass index, underweight, overweight, and obesity from 1975 to 2016: a pooled analysis of 2416 population-based measurement studies in 128.9 million children, adolescents, and adults. Lancet. 2017;390:2627–42. 29029897 10.1016/S0140-6736(17)32129-3 PMC5735219

[3] Collaboration NCDRF. Trends in adult body-mass index in 200 countries from 1975 to 2014: a pooled analysis of 1698 population-based measurement studies with 19.2 million participants. Lancet. 2016;387:1377–96. 27115820 10.1016/S0140-6736(16)30054-X PMC7615134

[4] Bray GA Heisel WE Afshin A. The science of obesity management: an endocrine society scientific statement. Endocr Rev. 2018;39:79–132. 29518206 10.1210/er.2017-00253 PMC5888222

[5] Flores-Dorantes MT Diaz-Lopez YE Gutierrez-Aguilar R. Environment and gene association with obesity and their impact on neurodegenerative and neurodevelopmental diseases. Front Neurosci. 2020;14:863. 32982666 10.3389/fnins.2020.00863 PMC7483585

[6] Sui SX Pasco JA. Obesity and brain function: the brain-body crosstalk. Medicina (Kaunas). 2020;56:499. 32987813 10.3390/medicina56100499 PMC7598577

[7] Han YP Tang X Han M. Relationship between obesity and structural brain abnormality: accumulated evidence from observational studies. Ageing Res Rev. 2021;71:101445. 34391946 10.1016/j.arr.2021.101445

[8] Smith E Hay P Campbell L Trollor JN. A review of the association between obesity and cognitive function across the lifespan: implications for novel approaches to prevention and treatment. Obes Rev. 2011;12:740–55. 21991597 10.1111/j.1467-789X.2011.00920.x

[9] Tanaka H Gourley DD Dekhtyar M Haley AP. Cognition, brain structure, and brain function in individuals with obesity and related disorders. Curr Obes Rep. 2020;9:544–9. 33064270 10.1007/s13679-020-00412-y

[10] Liu Y Basty N Whitcher B. Genetic architecture of 11 organ traits derived from abdominal MRI using deep learning. Elife. 2021;10:e65554. 34128465 10.7554/eLife.65554 PMC8205492

[11] Pulit SL Stoneman C Morris AP. Meta-analysis of genome-wide association studies for body fat distribution in 694 649 individuals of European ancestry. Hum Mol Genet. 2019;28:166–74. 30239722 10.1093/hmg/ddy327 PMC6298238

[12] Shungin D Winkler TW Croteau-Chonka DC. New genetic loci link adipose and insulin biology to body fat distribution. Nature. 2015;518:187–96. 25673412 10.1038/nature14132 PMC4338562

[13] Berndt SI Gustafsson S Magi R. Genome-wide meta-analysis identifies 11 new loci for anthropometric traits and provides insights into genetic architecture. Nat Genet. 2013;45:501–12. 23563607 10.1038/ng.2606 PMC3973018

[14] Bradfield JP Taal HR Timpson NJ. A genome-wide association meta-analysis identifies new childhood obesity loci. Nat Genet. 2012;44:526–31. 22484627 10.1038/ng.2247 PMC3370100

[15] Mishra A Malik R Hachiya T. Stroke genetics informs drug discovery and risk prediction across ancestries. Nature. 2022;611:115–23. 36180795 10.1038/s41586-022-05165-3 PMC9524349

[16] Jansen IE Savage JE Watanabe K. Genome-wide meta-analysis identifies new loci and functional pathways influencing Alzheimer’s disease risk. Nat Genet. 2019;51:404–13. 30617256 10.1038/s41588-018-0311-9 PMC6836675

[17] Iacoangeli A Lin T Khleifat A A. Genome-wide meta-analysis finds the ACSL5-ZDHHC6 locus is associated with ALS and links weight loss to the disease genetics. Cell Rep. 2020;33:108323. 33113361 10.1016/j.celrep.2020.108323 PMC7610013

[18] International Multiple Sclerosis Genetics Consortium. Multiple sclerosis genomic map implicates peripheral immune cells and microglia in susceptibility. Science. 2019;365:eaav7188. 31604244 10.1126/science.aav7188 PMC7241648

[19] Chia R Sabir MS Bandres-Ciga S. Genome sequencing analysis identifies new loci associated with Lewy body dementia and provides insights into its genetic architecture. Nat Genet. 2021;53:294–303. 33589841 10.1038/s41588-021-00785-3 PMC7946812

[20] Nagel M Watanabe K Stringer S Posthuma D van der Sluis S. Item-level analyses reveal genetic heterogeneity in neuroticism. Nat Commun. 2018;9:905. 29500382 10.1038/s41467-018-03242-8 PMC5834468

[21] Guo B Wang C Zhu Y. Causal associations of brain structure with bone mineral density: a large-scale genetic correlation study. Bone Res. 2023;11:37. 37474577 10.1038/s41413-023-00270-z PMC10359275

[22] Consortium GT. The GTEx Consortium atlas of genetic regulatory effects across human tissues. Science. 2020;369:1318–30. 32913098 10.1126/science.aaz1776 PMC7737656

[23] Gusev A Ko A Shi H. Integrative approaches for large-scale transcriptome-wide association studies. Nat Genet. 2016;48:245–52. 26854917 10.1038/ng.3506 PMC4767558

[24] Wen J Zhang J Zhang H. Large-scale genome-wide association studies reveal the genetic causal etiology between air pollutants and autoimmune diseases. J Transl Med. 2024;22:392. 38685026 10.1186/s12967-024-04928-y PMC11057084

[25] Zhang J Wen J Dai Z. Causal association and shared genetics between telomere length and COVID-19 outcomes: new evidence from the latest large-scale summary statistics. Comput Struct Biotechnol J. 2024;23:2429–41. 38882679 10.1016/j.csbj.2024.05.012 PMC11176559

[26] Bowden J Smith GD Burgess S. Mendelian randomization with invalid instruments: effect estimation and bias detection through Egger regression. Int J Epidemiol. 2015;44:512–25. 26050253 10.1093/ije/dyv080 PMC4469799

[27] Bowden J Del Greco M F Minelli C. Improving the accuracy of two-sample summary-data mendelian randomization: moving beyond the NOME assumption. Int J Epidemiol. 2019;48:728–42. 30561657 10.1093/ije/dyy258 PMC6659376

[28] Kim MS Kim WJ Khera AV. Association between adiposity and cardiovascular outcomes: an umbrella review and meta-analysis of observational and Mendelian randomization studies. Eur Heart J. 2021;42:3388–403. 34333589 10.1093/eurheartj/ehab454 PMC8423481

[29] Horn JW Feng T Morkedal B. Obesity and risk for first ischemic stroke depends on metabolic syndrome: the HUNT Study. Stroke. 2021;52:3555–61. 34281375 10.1161/STROKEAHA.120.033016

[30] Kim ID Cave JW Cho S. Aflibercept, a VEGF (Vascular Endothelial Growth Factor)-trap, reduces vascular permeability and stroke-induced brain swelling in obese mice. Stroke. 2021;52:2637–48. 34192895 10.1161/STROKEAHA.121.034362 PMC8312568

[31] Mina T Yew YW Ng HK. Adiposity impacts cognitive function in Asian populations: an epidemiological and Mendelian randomization study. Lancet Reg Health West Pac. 2023;33:100710. 36851942 10.1016/j.lanwpc.2023.100710 PMC9957736

[32] Norris T Salzmann A Henry A Garfield V Pereira SMP. The relationship between adiposity and cognitive function: a bidirectional Mendelian randomization study in UK Biobank. Int J Epidemiol. 2023;52:1074–85. 37029912 10.1093/ije/dyad043 PMC10396406

[33] Chen W Feng J Guo J. Obesity causally influencing brain cortical structure: a Mendelian randomization study. Cereb Cortex. 2023;33:9409–16. 37328935 10.1093/cercor/bhad214

[34] Feng Z Fang C Ma Y Chang J. Obesity-induced blood-brain barrier dysfunction: phenotypes and mechanisms. J Neuroinflammation. 2024;21:110. 38678254 10.1186/s12974-024-03104-9 PMC11056074

[35] Le Thuc O Garcia-Caceres C. Obesity-induced inflammation: connecting the periphery to the brain. Nat Metab. 2024;6:1237–52. 38997442 10.1038/s42255-024-01079-8

[36] Chen KE Lainez NM Nair MG Coss D. Visceral adipose tissue imparts peripheral macrophage influx into the hypothalamus. J Neuroinflammation. 2021;18:140. 34154608 10.1186/s12974-021-02183-2 PMC8218389

[37] Vander Velden JW Osborne DM. Prolonged diet-induced obesity modifies DNA methylation and gene expression in the hippocampus. Neurosci Lett. 2022;780:136656. 35469824 10.1016/j.neulet.2022.136656

