# Supplementary material for: Investigating multidimensional associations between obesity-related traits and brain health, and identifying potential mechanisms
Source: Medicine (Baltimore). 2026 Jan 9;105(2):e46831. doi: 10.1097/MD.0000000000046831 (PMC12795085; doi:10.1097/MD.0000000000046831)
Supplement: Supplementary file 2 [file medi-105-e46831-s002.pdf]

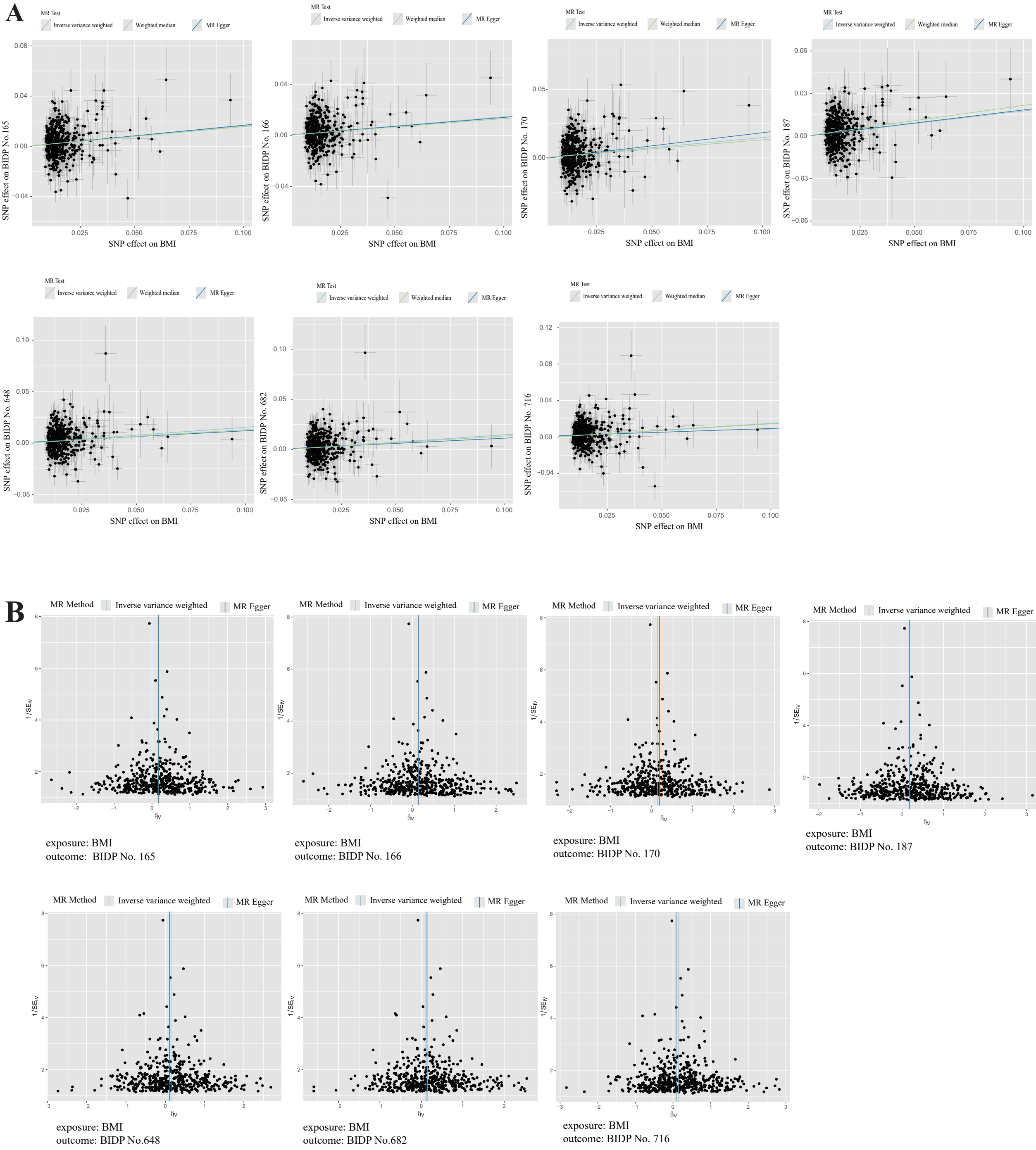
A
MR Test
Inverse variance weighted
Weighted median
MR Egger
SNP effect on BIDP No.165
SNP effect on BIDP No. 166
SNP effect on BIDP No. 170
SNP effect on BIDP No. 187
SNP effect on BIDP No. 648
SNP effect on BIDP No. 682
SNP effect on BIDP No. 716
SNP effect on BMI
B
MR Method
Inverse variance weighted
MR Egger
exposure: BMI
outcome: BIDP No. 165
exposure: BMI
outcome: BIDP No. 166
exposure: BMI
outcome: BIDP No. 170
exposure: BMI
outcome: BIDP No. 187
exposure: BMI
outcome: BIDP No.648
exposure: BMI
outcome: BIDP No.682
exposure: BMI
outcome: BIDP No. 716

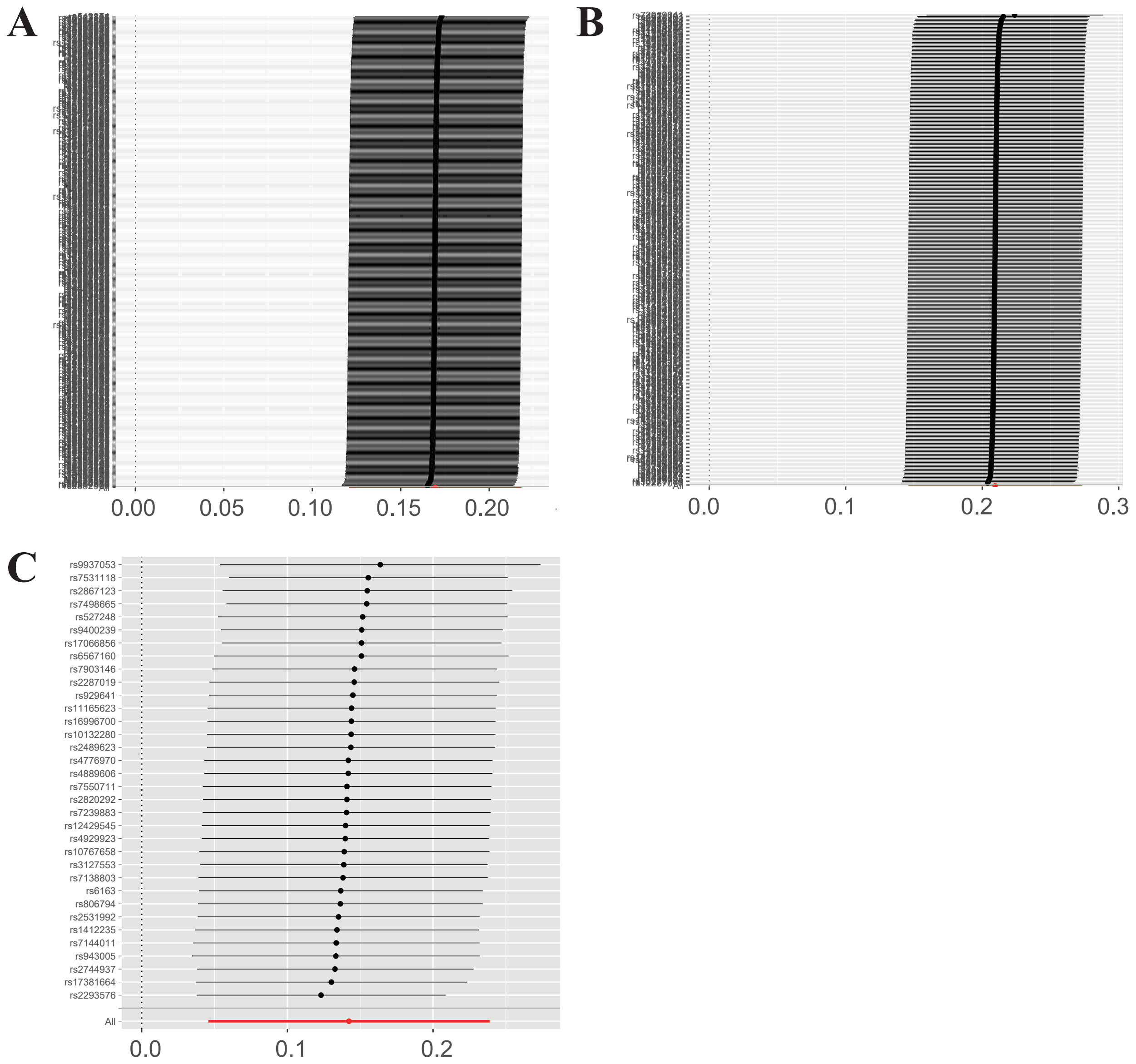
A
All
0.00
0.05
0.10
0.15
0.20
B
All
0.0
0.1
0.2
0.3
C
rs9937053
rs7531118
rs2867123
rs7498665
rs527248
rs9400239
rs17066856
rs6567160
rs7903146
rs2287019
rs929641
rs11165623
rs16996700
rs10132280
rs2489623
rs4776970
rs4889606
rs7550711
rs2820292
rs7239883
rs12429545
rs4929923
rs10767658
rs3127553
rs7138803
rs6163
rs806794
rs2531992
rs1412235
rs7144011
rs943005
rs2744937
rs17381664
rs2293576
All
0.0
0.1
0.2

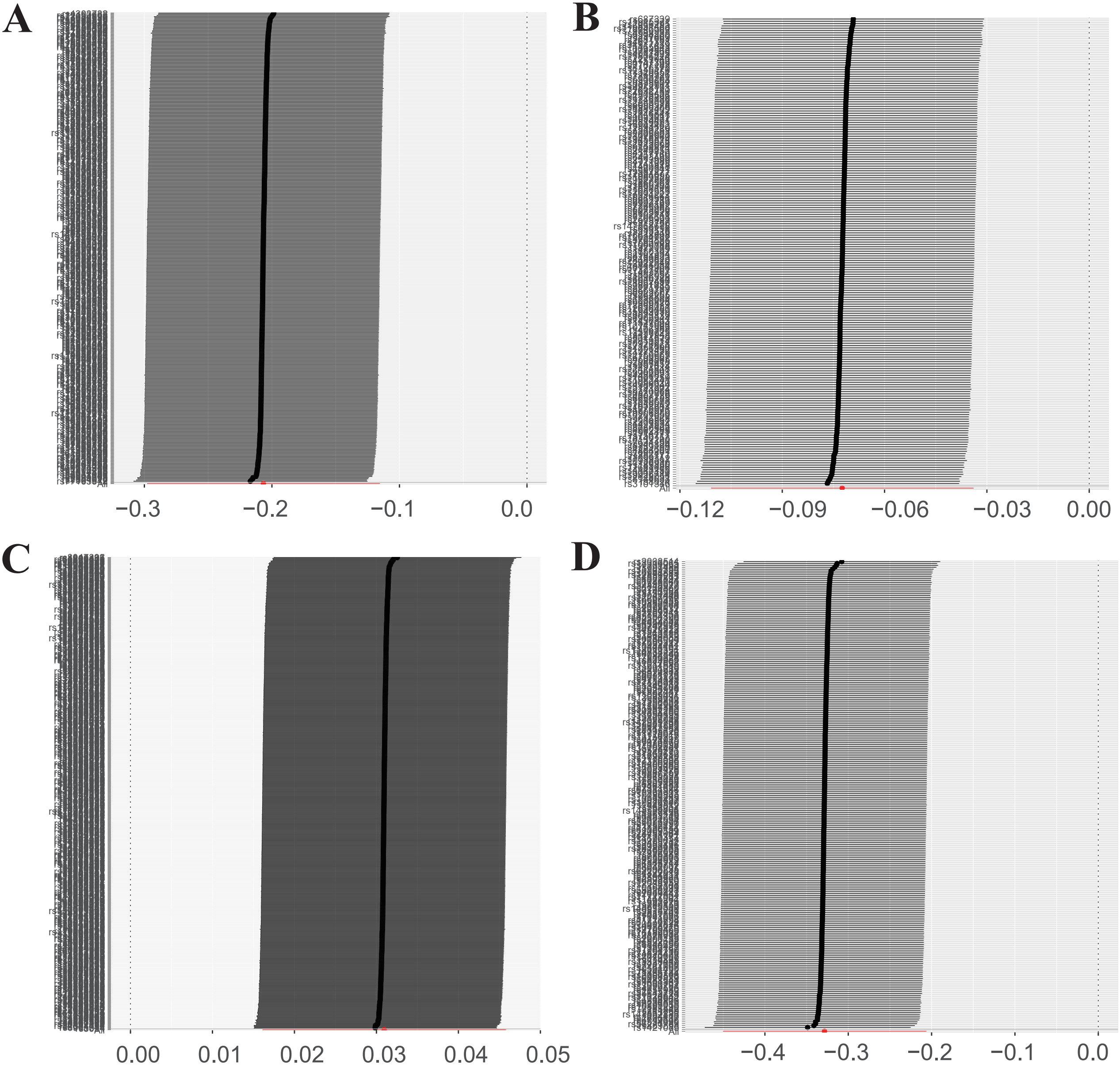
A
−0.3
−0.2
−0.1
0.0
B
−0.12
−0.09
−0.06
−0.03
0.00
C
0.00
0.01
0.02
0.03
0.04
0.05
D
−0.4
−0.3
−0.2
−0.1
0.0

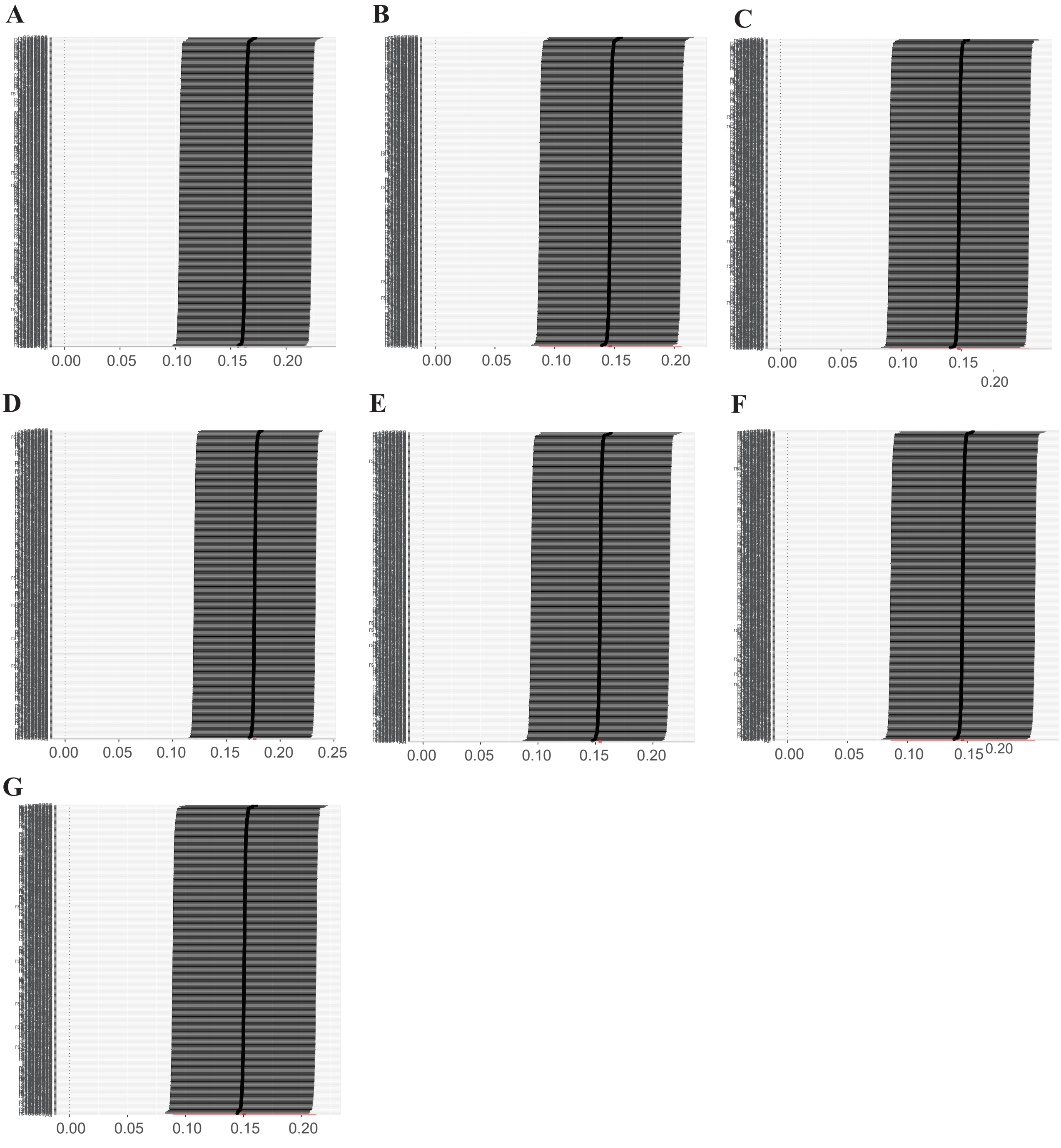
A
0.00
0.05
0.10
0.15
0.20
B
0.00
0.05
0.10
0.15
0.20
C
0.00
0.05
0.10
0.15
0.20
D
0.00
0.05
0.10
0.15
0.20
0.25
E
0.00
0.05
0.10
0.15
0.20
F
0.00
0.05
0.10
0.15
0.20
G
0.00
0.05
0.10
0.15
0.20

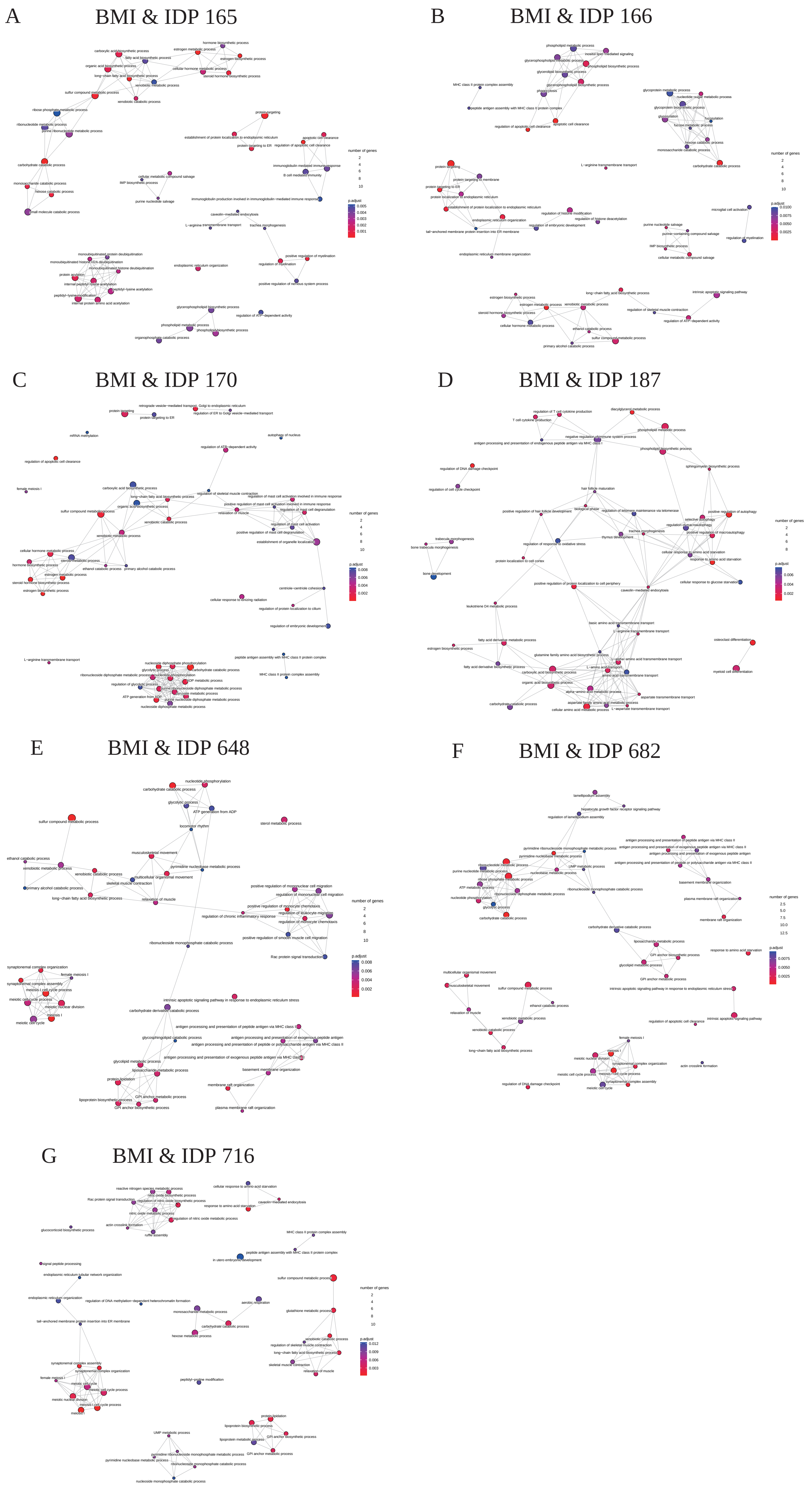
A
BMI & IDP 165
B
BMI & IDP 166
C
BMI & IDP 170
D
BMI & IDP 187
E
BMI & IDP 648
F
BMI & IDP 682
G
BMI & IDP 716
number of genes
p.adjust

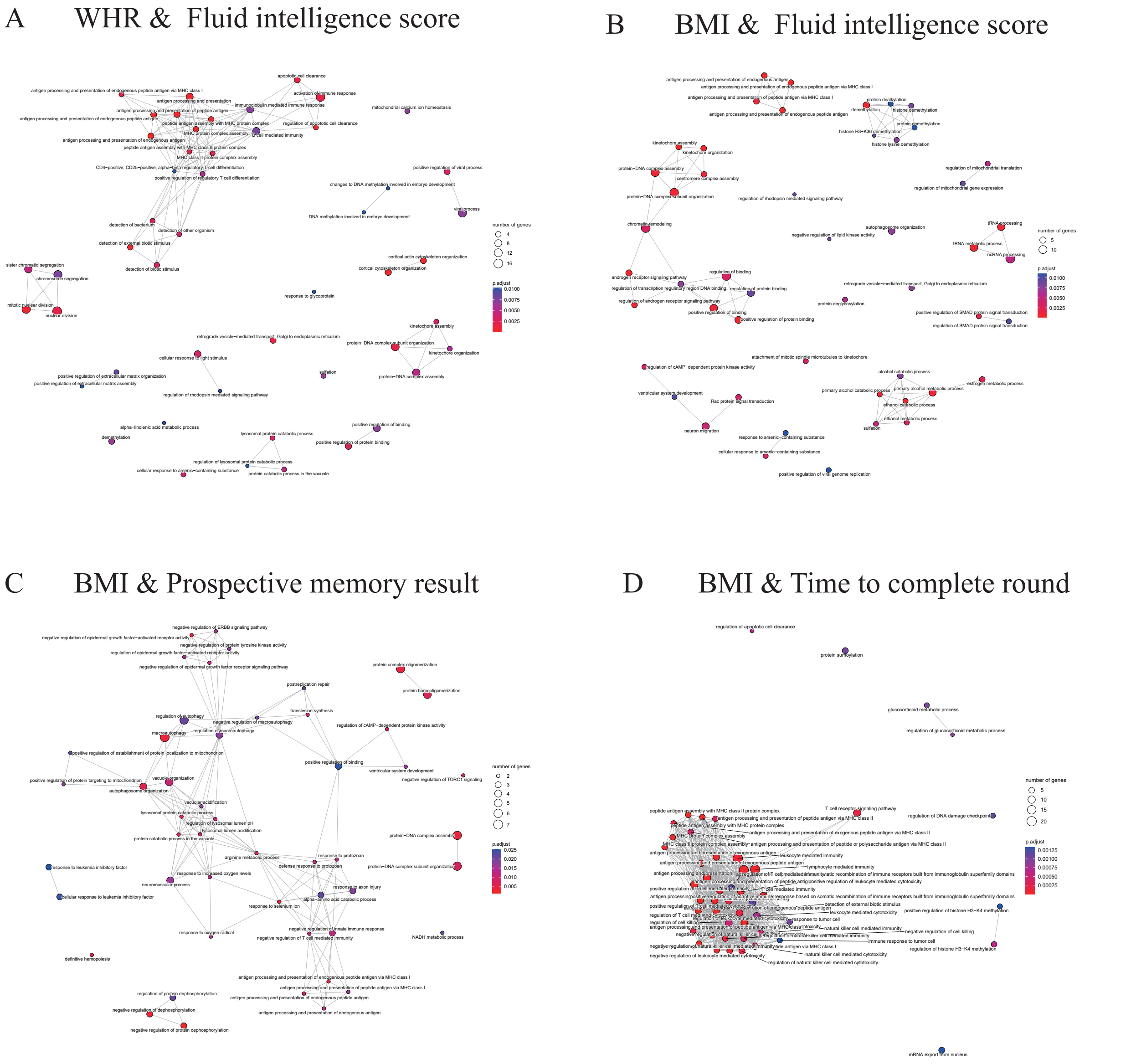
A WHR & Fluid intelligence score
apoptotic cell clearance
activation of immune response
antigen processing and presentation of endogenous peptide antigen via MHC class I
antigen processing and presentation
immunoglobulin mediated immune response
mitochondrial calcium ion homeostasis
antigen processing and presentation of peptide antigen
antigen processing and presentation of endogenous peptide antigen
peptide antigen assembly with MHC protein complex
regulation of apoptotic cell clearance
MHC protein complex assembly
B cell mediated immunity
antigen processing and presentation of endogenous antigen
peptide antigen assembly with MHC class II protein complex
MHC class II protein complex assembly
CD4−positive, CD25−positive, alpha−beta regulatory T cell differentiation
positive regulation of regulatory T cell differentiation
positive regulation of viral process
changes to DNA methylation involved in embryo development
viral process
DNA methylation involved in embryo development
detection of bacterium
detection of other organism
detection of external biotic stimulus
detection of biotic stimulus
number of genes
4
8
12
16
sister chromatid segregation
chromosome segregation
cortical actin cytoskeleton organization
cortical cytoskeleton organization
p.adjust
0.0100
0.0075
0.0050
0.0025
response to glycoprotein
mitotic nuclear division
nuclear division
kinetochore assembly
retrograde vesicle−mediated transport, Golgi to endoplasmic reticulum
protein−DNA complex subunit organization
kinetochore organization
cellular response to light stimulus
sulfation
protein−DNA complex assembly
positive regulation of extracellular matrix organization
positive regulation of extracellular matrix assembly
regulation of rhodopsin mediated signaling pathway
alpha−linolenic acid metabolic process
positive regulation of binding
lysosomal protein catabolic process
demethylation
positive regulation of protein binding
regulation of lysosomal protein catabolic process
cellular response to arsenic−containing substance
protein catabolic process in the vacuole
B BMI & Fluid intelligence score
antigen processing and presentation of endogenous antigen
antigen processing and presentation of endogenous peptide antigen via MHC class I
antigen processing and presentation of peptide antigen via MHC class I
antigen processing and presentation of endogenous peptide antigen
protein dealkylation
demethylation
histone demethylation
protein demethylation
histone H3−K36 demethylation
histone lysine demethylation
kinetochore assembly
kinetochore organization
protein−DNA complex assembly
centromere complex assembly
regulation of mitochondrial translation
regulation of mitochondrial gene expression
protein−DNA complex subunit organization
regulation of rhodopsin mediated signaling pathway
chromatin remodeling
autophagosome organization
tRNA processing
negative regulation of lipid kinase activity
tRNA metabolic process
ncRNA processing
number of genes
5
10
androgen receptor signaling pathway
regulation of binding
p.adjust
0.0100
0.0075
0.0050
0.0025
regulation of transcription regulatory region DNA binding
regulation of protein binding
retrograde vesicle−mediated transport, Golgi to endoplasmic reticulum
regulation of androgen receptor signaling pathway
protein deglycosylation
positive regulation of binding
positive regulation of protein binding
positive regulation of SMAD protein signal transduction
regulation of SMAD protein signal transduction
attachment of mitotic spindle microtubules to kinetochore
regulation of cAMP−dependent protein kinase activity
alcohol catabolic process
primary alcohol catabolic process
primary alcohol metabolic process
estrogen metabolic process
ventricular system development
Rac protein signal transduction
ethanol catabolic process
ethanol metabolic process
sulfation
neuron migration
response to arsenic−containing substance
cellular response to arsenic−containing substance
positive regulation of viral genome replication
C BMI & Prospective memory result
negative regulation of ERBB signaling pathway
negative regulation of epidermal growth factor−activated receptor activity
negative regulation of protein tyrosine kinase activity
regulation of epidermal growth factor−activated receptor activity
negative regulation of epidermal growth factor receptor signaling pathway
protein complex oligomerization
protein homooligomerization
postreplication repair
translesion synthesis
regulation of autophagy
negative regulation of macroautophagy
regulation of cAMP−dependent protein kinase activity
macroautophagy
regulation of macroautophagy
positive regulation of establishment of protein localization to mitochondrion
positive regulation of binding
ventricular system development
negative regulation of TORC1 signaling
positive regulation of protein targeting to mitochondrion
vacuole organization
autophagosome organization
number of genes
2
3
4
5
6
7
vacuolar acidification
lysosomal protein catabolic process
regulation of lysosomal lumen pH
lysosomal lumen acidification
protein catabolic process in the vacuole
protein−DNA complex assembly
p.adjust
0.025
0.020
0.015
0.010
0.005
response to protozoan
arginine metabolic process
defense response to protozoan
protein−DNA complex subunit organization
response to leukemia inhibitory factor
response to increased oxygen levels
neuromuscular process
response to axon injury
alpha−amino acid catabolic process
cellular response to leukemia inhibitory factor
response to selenium ion
negative regulation of innate immune response
response to oxygen radical
negative regulation of T cell mediated immunity
NADH metabolic process
definitive hemopoiesis
antigen processing and presentation of endogenous peptide antigen via MHC class I
antigen processing and presentation of peptide antigen via MHC class I
antigen processing and presentation of endogenous peptide antigen
regulation of protein dephosphorylation
antigen processing and presentation of endogenous antigen
negative regulation of dephosphorylation
negative regulation of protein dephosphorylation
D BMI & Time to complete round
regulation of apoptotic cell clearance
protein sumoylation
glucocorticoid metabolic process
regulation of glucocorticoid metabolic process
number of genes
5
10
15
20
peptide antigen assembly with MHC class II protein complex
T cell receptor signaling pathway
antigen processing and presentation of peptide antigen via MHC class II
regulation of DNA damage checkpoint
peptide antigen assembly with MHC protein complex
MHC protein complex assembly
antigen processing and presentation of exogenous peptide antigen via MHC class II
MHC class II protein complex assembly
antigen processing and presentation of peptide or polysaccharide antigen via MHC class II
p.adjust
0.00125
0.00100
0.00075
0.00050
0.00025
antigen processing and presentation of exogenous antigen
leukocyte mediated immunity
antigen processing and presentation of exogenous peptide antigen
lymphocyte mediated immunity
antigen processing and presentation
adaptive immune response based on somatic recombination of immune receptors built from immunoglobulin superfamily domains
positive regulation of leukocyte mediated cytotoxicity
T cell mediated immunity
positive regulation of adaptive immune response based on somatic recombination of immune receptors built from immunoglobulin superfamily domains
detection of external biotic stimulus
leukocyte mediated cytotoxicity
positive regulation of histone H3−K4 methylation
response to tumor cell
natural killer cell mediated immunity
negative regulation of cell killing
immune response to tumor cell
regulation of histone H3−K4 methylation
natural killer cell mediated cytotoxicity
negative regulation of leukocyte mediated cytotoxicity
regulation of natural killer cell mediated cytotoxicity
mRNA export from nucleus
